# Highly Efficient and Controllable Methodology of the Cd_0.25_Zn_0.75_Se/ZnS Core/Shell Quantum Dots Synthesis

**DOI:** 10.3390/nano11102616

**Published:** 2021-10-05

**Authors:** Liudmila Loghina, Maksym Chylii, Anastasia Kaderavkova, Stanislav Slang, Petr Svec, Jhonatan Rodriguez Pereira, Bozena Frumarova, Miroslav Cieslar, Miroslav Vlcek

**Affiliations:** 1 Center of Materials and Nanotechnologies, Faculty of Chemical Technology, University of Pardubice, 53002 Pardubice, Czech Republic; Maksym.Chylii@upce.cz (M.C.); Anastasia.Kaderavkova@upce.cz (A.K.); Stanislav.Slang@upce.cz (S.S.); Jhonatan.RodriguezPereira@upce.cz (J.R.P.); Bozena.Frumarova@upce.cz (B.F.); Miroslav.Vlcek@upce.cz (M.V.); 2 Department of General and Inorganic Chemistry, Faculty of Chemical Technology, University of Pardubice, 53210 Pardubice, Czech Republic; petr.svec2@upce.cz; 3 Faculty of Mathematics and Physics, Charles University, 12116 Prague, Czech Republic; cieslar@met.mff.cuni.cz

**Keywords:** core/shell quantum dots, controllable synthesis, methodology, non-toxic chalcogen source

## Abstract

The surface of any binary or multi-component nanocrystal has imperfections and defects. The number of surface defects depends both on the nature of the nanomaterial and on the method of its preparation. One of the possibilities to confine the number of surface defects is the epitaxial growth of the shell, which leads to a change in the physical properties while maintaining the morphology of the core. To form a shell of the desired thickness, an accurate calculation of the amount of its precursors is substantial to avoid the appearance of individual crystals consisting of the shell material. This study aimed to develop an effective calculation method for the theoretical amount of precursors required for the formation of a ZnS shell on the surface of a Cd_0.25_Zn_0.75_Se core, followed by the practical implementation of theoretical calculations and characterization of the prepared nanomaterials. This method allows the complete control of the masses and volumes of the initial reagents, which will in turn prevent undesirable nucleation of nuclei consisting of the shell material. In the synthesis of Cd_0.25_Zn_0.75_Se/ZnS core/shell quantum dots (QDs), the sources of chalcogens were substituted seleno- and thioureas, which are capable of not only supplanting modern toxic sources of sulfur and selenium but also allowing one to perform the controlled synthesis of highly photoluminescent QDs with a low number of surface defects. The result of this shell overcoating method was an impetuous augmentation in the photoluminescence quantum yield (PL QY up to 83%), uniformity in size and shape, and a high yield of nanomaterials. The developed synthetic technique of core/shell QDs provides a controlled growth of the shell on the core surface, which makes it possible to transfer this method to an industrial scale.

## 1. Introduction

Over the past decades, ternary semiconductor quantum dots (QDs) have drawn vast scientific and technological interest due to their unique size-, shape-, composition-dependent physical, chemical, and optoelectronics properties [1,2,3]. The technological importance of such QDs (CdZnS, CdZnSe, CdSSe, etc.) is determined by the tunability of their emission and absorbance features, which opens up wide possibilities for applications in diverse fields such as light-emitting diodes (LEDs) [4], solar cells [5], alpha, gamma, and neutron scintillators [6,7,8], catalysts for reaction processes [9,10,11], and bio labeling [12]. In particular, CdZnSe QDs have attracted research focus because of the possibility to adjust the compositional Cd:Zn ratio and thus to tune the emission color over the whole visible spectrum [13,14]. Another successful synthetic strategy for the preparation of highly photoluminescent Cd-Zn-Se QDs with PL QYs up to 64% by using (Z)-1-hexyl-3-(octadec-9-enyl)selenourea as a new selenium precursor was recently presented [15].

However, ternary semiconductor QDs suffer from poor stability against the surrounding environment due to the surface defects that act as nonradiative recombination sites [16,17]. Such a phenomenon leads to the degradation of PL QY and hinders the actual application of QDs. Growing a mono- or multilayer shell on the QD’s core is one of the effective approaches to solve this problem. Usually, the outer shell is a wide-bandgap semiconductor material that provides effective elimination of surface-related defects in the core QDs, which leads to passivation of the surface, improved photostability, better dispersibility [18,19,20,21], suppressed Forster resonance energy transfer (FRET), and enhanced optical characteristics (PL QY, emission wavelength, and carrier lifetime) of QDs. These properties are essential for the future commercial applications of core/shell QDs. Recently, the research focus turned to the ternary core/shell systems with different shell compositions, such as CdSeS/ZnS [22,23], CdZnSe/ZnSe [24,25,26], CdTeSe/ZnSe [27,28], and CdZnS/ZnS [29,30] QDs. It is important to note that to achieve the desired optical characteristics and an increase in PL QY, careful selection of the appropriate composition of both core and shell QDs (considering their bandgap and lattice mismatch) and also the thickness of the shell is required. Until now, several strategies for the synthesis of core/shell ternary QDs have been reported. Fitzmorris et al. [31] introduced a synthetic approach that greatly enhanced the PL QY (from 9.6% for CdZnSe QDs core to 42% for Cd_1−x_Zn_x_Se/ZnSe and 63% for Cd_1−x_Zn_x_Se/ZnSe/ZnS) of Cd_1−x_Zn_x_Se/ZnSe/ZnS after a shell growth. The CdZnSe core was prepared at 320 °C/1 h in one step without any purification process using oleic acid and oleylamine as surface ligands; then, ZnSe and ZnS shells were formed by the successive injection of precursors (TOP-Se and TOP-S) in one-pot at 240 °C and 200 °C, respectively.

Later, Zhang et al. [32] prepared CdZnSe/ZnSe quantum dots via the modified one-pot method [31]. CdZnSe QDs were synthesized at 290–310 °C/8 min with oleic acid and without oleylamine. Then, the reaction mixture was heated to 300–320 °C and the Se-TBP precursor was slowly injected. By varying the reaction temperatures and Se-TBP amount (1–2.5 mmol) within the shell formation, the absolute PL QY increased from 20% to 50%.

Susumu et al. [33] reported a series of Cd_x_Zn_1−x_Se/Cd_y_Zn_1−y_S/ZnS and ZnSe/Cd_y_Zn_1−y_S/ZnS QDs prepared from ZnSe cores (synthesized from diethylzinc and Se-TOP in oleylamine solution at 290 °C) with a subsequent surface modification by ligand exchange with hydrophilic compact ligands. Cd_x_Zn_1−x_Se cores were formed via cation exchange with Cd^2+^ ions from as-prepared ZnSe QDs in 1-octadecene (ODE), oleylamine, TOP, and dodecylphosphonic acid solution at 220 °C. Following that, overcoating with Cd_y_Zn_1−y_S and ZnS shells was conducted in the same flask by adding Zn and Cd oleate solutions and a ~1.2–1.5-fold excess of *n*-octanethiol (with respect to metal precursors) at 290–310 °C for 4–6 h. The PL QY of the prepared core/shell Cd_x_Zn_1−x_Se/Cd_y_Zn_1−y_S/ZnS QDs enhanced from 1–2% after cation exchange to 53%.

Recently, Jin et al. [24] presented a cation exchange-assisted method for the preparation of ZnCdSe/ZnSe core/shell QDs. The typical synthesis was carried out by employing CdO, zinc acetate, oleic acid, and ODE at 300 °C with an injection of Se-TOP precursor. After 12 min of the reaction process, an additional amount of Se-TOP in ODE was dripped to the solution. The shell formation took 30 min. The resulting core/shell ZnCdSe/ZnSe (8 monolayers) QDs reached an absolute PL QY of 99%. In addition, the authors reported a detailed calculation for the amount of shell precursors required to form a different number of monolayers.

Despite the variety of proposed methods, it remains a challenging task to synthesize CdZnSe cores with an appropriate shell thickness and high PL QY using nontoxic sources of S and Se.

Herein, we successfully elaborated on an efficient way of shell growth on the already formed QDs core. The presented method is based on the calculated ratio of the volume/mass of the core to the shell. Gram-scale synthesis of the Cd_0.25_Zn_0.75_Se core (3.5 g) was carried out by a hot-injection method followed by the reaction with a calculated amount of cadmium and zinc linoleates with (*Z*)-*N*-(octadec-9-enyl)morpholine-4-carboselenoamide (*N*,*N*′,*N*′-trisubstituted selenourea), which was not previously used as a source of selenium. The shell, consisting of 1–5 monolayers (MLs) of zinc sulfide, grew on the core upon precisely dosed injection of a calculated amount of (*Z*)-1-(octadec-9-enyl)-3-phenylthiourea dissolved in a mixture of oleylamine and 1-octadecene at 240 °C, to a diluted solution of a half-fold excess of zinc linoleate and the core. The dosed injection of the sulfur source avoids the parallel formation of zinc sulfide cores and ensures uniform shell formation. The presented method for calculating the amount of materials, required for a shell growth of the desired thickness, makes possible the application of this technique to the synthesis of core/shell QDs of any composition and size. In our opinion, the availability of precursors, as well as the simplicity and reproducibility of calculations of the materials required to grow a shell of the desired thickness, makes the proposed gram-scale method promising for the widespread production of LEDs and bio-, light-, and chemical sensors.

## 2. Materials and Methods

### 2.1. Materials

Cadmium oxide (CdO, 99.99%), zinc oxide (ZnO, 99%), 1-octadecene (ODE, technical grade, 90%), linoleic acid (LA, technical grade 60–74%), oleylamine (OAm, technical grade, 90%), selenium (Se, 99.999%), phenyl isothiocyanate (PhNCS, 98%), morpholine (99%), chloroform-d (CDCl_3_, 99.8 atom % D), and silica gel (high-purity grade, pore size 60 Å, 230–400 mesh particle size) were purchased from Sigma-Aldrich and used without further purification. Sodium hydroxide (NaOH, p.a.) and solvents were purchased from Fisher Scientific and used for the purification of organic precursors and QDs. Triethylamine (Et_3_N, p.a.) and tetrahydrofuran (THF, p.a.) were purified by distillation over NaOH and sodium correspondingly prior to the synthesis. Syntheses of QDs were carried out using standard Schlenk techniques under an inert atmosphere.

### 2.2. Synthesis of Selenium and Sulphur Precursors

#### 2.2.1. Synthesis of (Z)-1-isocyanooctadec-9-ene

Oleylamine (OAm, 0.01 mol) in 40 mL of CH_2_Cl_2_ and CHCl_3_ (0.11 mol) were added to a freshly prepared 50% NaOH solution (22.80 g, 0.57 mol). After that, a phase transfer catalyst (tetraethylammonium bromide, 0.005 mol) was added to the resulting mixture mechanically stirred at room temperature. Afterward, the reaction mixture was stirred under reflux for 24 h. The synthesis was controlled by thin-layer chromatography (TLC) (CH_2_Cl_2_). The resulting reaction mixture was poured into 100 mL of water and then extracted with CH_2_Cl_2_ (3 × 50 mL). The combined organic extracts were washed with aqueous NH_4_Cl (sat.), brine, and then dried over anhydrous Na_2_SO_4_. After the solvent evaporation, the residue was purified by flash chromatography (CH_2_Cl_2_). A colorless liquid was obtained in a 63% yield.

^1^H NMR (500.13 MHz, CDCl_3_), ppm: 5.27–5.37 (m, 2H, -CH=CH-), 3.34–3.36 (m, 2H, CH_2_), 1.98–2.03 (m, 2H, CH_2_), 1.62–1.69 (m, 2H, CH_2_), 1.39–1.45 (m, 2H, CH_2_), 1.25–1.30 (m, 22H, 11 × CH_2_), 0.85–0.89 (t, 3H, CH_3_).

^13^C NMR (125.76 MHz, CDCl_3_), ppm: 155.9 (N≡C), 130.0, (*C*H=CH), 129.7 (CH=*C*H), 41.50 (m, CH_2_N≡C), 32.0, 29.8, 29.7, 29.6, 29.5, 29.4, 29.3, 29.2, 29.1, 28.7, 27.3, 27.2, 26.3, 22.7, 14.2.

#### 2.2.2. Synthesis of (Z)-1-isoselenocyanatooctadec-9-ene

Dry Et_3_N (0.026 mol) and (*Z*)-1-isocyanooctadec-9-ene (0.026 mol) in 50 mL of dry THF were mixed in a reaction flask. The mixture was degassed and selenium powder (0.013 mol) was added under Ar. The reaction mixture was heated to reflux for 8 h under Ar and then naturally cooled to room temperature. Afterward, the second portion of selenium was added (0.013 mol) and heated until the complete dissolution of selenium. The resulting clear solution was concentrated, followed by purification on silica gel using petroleum ether as an eluent. A yellowish liquid product was obtained in 88% yield.

^1^H NMR (500.13 MHz, CDCl_3_), ppm: 5.27–5.35 (m, 2H, -CH = CH-), 3.57 (t, 2H, *J* = 6.7 Hz, -CH_2_-NCSe), 1.93–1.99 (m, 2H, CH_2_), 1.67–1.73 (m, 2H, CH_2_), 1.36–1.42 (m, 2H, CH_2_), 1.23–1.28 (m, 22H, 11 × CH_2_), 0.85 (t, 3H, *J* = 6.7 Hz, CH_3_).

^13^C NMR (125.76 MHz, CDCl_3_), ppm: 130.0 (*C*H = CH), 129.7 (CH = *C*H), 122.0 (N = C = Se), 45.5 (br, *C*H_2_NCSe), 32.4 (m), 29.9, 29.7, 29.6, 29.5, 29.4, 29.3, 29.2, 29.0, 28.8, 27.3, 27.2, 26.5, 22.7, 14.2. ^77^Se NMR (95.35 MHz, CDCl_3_), ppm: −358.3.

#### 2.2.3. Synthesis of (Z)-*N*-(octadec-9-enyl)morpholine-4-carboselenoamide

To a solution of (*Z*)-1-isoselenocyanatooctadec-9-ene (0.02 mol) in 100 mL of CH_2_Cl_2_, morpholine (0.02 mol) was added. The reaction mixture was stirred for 1 h without any exposure to light. The reaction was monitored by TLC (CH_2_Cl_2_). After the solvent evaporation, a light-yellow semisolid was collected. The reaction yield was quantitative.

^1^H NMR (500.13 MHz, CDCl_3_), ppm: 6.06 (br, 1H, NH), 5.26–5.32 (m, 2H, -CH = CH-), 3.81 (s, 4H, 2 × CH_2_), 3.64 (s, 6H, 3 × CH_2_), 1.87–1.96 (m, 2H, CH_2_), 1.52–1.61 (m, 4H, 2 × CH_2_), 1.16–1.26 (m, 22H, 11 × CH_2_), 0.79 (t, 3H, *J* = 6.84 Hz, CH_3_).

^13^C NMR (125.76 MHz, CDCl_3_), ppm: 181.9 (C = Se), 129.6 (2xCH = CH), 66.0, 49.2, 48.9, 31.7, 29.6, 29.4, 29.2, 29.1, 27.1, 26.8, 22.6, 14.0.

#### 2.2.4. Synthesis (Z)-1-(octadec-9-enyl)-3-phenylthiourea

To a solution of phenyl isothiocyanate (0.05 mol) in CH_2_Cl_2_ (75 mL), oleylamine (0.05 mol) was added dropwise with permanent stirring at room temperature (exothermic process). The reaction was stirred for one hour under the monitoring by TLC (CH_2_Cl_2_). After distilling off the solvent, the semisolid was obtained with quantitative yield.

^1^H NMR (500.13 MHz, CDCl_3_), ppm: 8.42 (br, 1H, NH), 7.40 (t, 2H, *J* = 7.8 Hz, Ph), 7.25–7.28 (m, 1H, Ph), 7.19–7.21 (d, 2H, *J* = 7.8 Hz, Ph), 6.08 (br, 1H, NH), 5.31–5.37 (m, 2H, -CH = CH-), 3.57–3.61 (m, 2H, CH_2_), 1.93–2.01 (m, 2H, CH_2_), 1.51–1.57 (m, 2H, CH_2_), 1.23–1.31 (m, 22H, 11 × CH_2_), 0.86 (t, 3H, *J* = 6.9 Hz, CH_3_).

^13^C NMR (125.76 MHz, CDCl_3_), ppm: 180.5 (C = S), 136.5 (Ph), 130.2 (Ph), 130.1 (CH = CH), 129.9 (CH = CH), 127.2 (Ph), 125.3 (Ph), 45.6 (CH_2_NH), 32.0, 29.9, 29.8, 29.7, 29.6, 29.5, 29.4, 29.3, 29.2, 29.1, 27.3, 27.2, 27.0, 22.8, 14.2.

### 2.3. Preparation of Cd_0.25_Zn_0.75_Se/ZnS (1–5 MLs) QDs

#### 2.3.1. Synthesis of Core Cd_0.25_Zn_0.75_Se

The reaction mixture consisting of CdO (0.005 mol), ZnO (0.015 mol), LA (0.06 mol), and 60 mL of ODE was degassed for 15–20 min at room temperature and then at 150 °C until complete homogenization. The temperature of the reaction mixture was then raised to 240 °C (the growth temperature) and a solution of (*Z*)-N-(octadec-9-enyl)morpholine-4-carboselenoamide (*SU*, 0.019 mol) in 9.4 mL of OAm and 1.6 mL of ODE was injected in one portion. All steps of the reaction process were carried out under vigorous stirring in an inert atmosphere. After the injection of the selenium precursor, the reaction mixture was further stirred for an additional 30 min while maintaining the growth temperature. The naturally cooled (40–45 °C) reaction mixture was diluted twofold with CHCl_3_, and QDs were precipitated using a mixture of acetone and methanol (20:1). The formed precipitate was separated by centrifugation (10.000 rpm, 7 min). The separated QDs were dissolved in a minimum amount of CHCl_3_ followed by precipitation with acetone. This procedure was carried out 3 more times to remove the starting components and solvent. The purified QDs were dried under vacuum for 5 h. The yield of core Cd_0.25_Zn_0.75_Se was 3.5 g.

To determine the amount of Cd_0.25_Zn_0.75_Se substance with a known mass of QDs stabilized by an organic shell, the chemical decomposition of the nanomaterial was carried out. Dried to the constant weight, Cd_0.25_Zn_0.75_Se QDs (0.200 g) were added in 10% HCl (20 mL) and stirred at room temperature for 3 h. The organic layer was extracted with diethyl ether (3 × 20 mL). The combined organic extracts were washed with brine and dried over Na_2_SO_4_. The ether extract was quantitatively filtered through a thin layer of silica gel. The mass of the residue obtained after distilling off the solvent was 0.031 g. Based on the performed procedure, it was found that 0.200 g of the nanomaterial contained 0.169 g of Cd_0.25_Zn_0.75_Se.

#### 2.3.2. Synthesis of Core/Shell Cd_0.25_Zn_0.75_Se/ZnS 1 ML QDs

The calculated amount of ZnO (0.00126 mol) was mixed with LA (1.57 mL) and ODE (4 mL) in a 50 mL Schlenk flask. The reaction mixture was degassed for 15–20 min at room temperature and then at 150 °C until complete homogenization. Then, the temperature was raised to 240 °C (the growth temperature) and the solution of Cd_0.25_Zn_0.75_Se QDs (0.196 g) in 2 mL of ODE was added by one portion. The calculated amount of (*Z*)-1-(octadec-9-enyl)-3-phenylthiourea (*TU*, 0.000615 mol) was dissolved in ODE (1.25 mL) and OAm (0.5 mL). This solution was added in portions (0.2 mL/2 min) under vigorous stirring (inert atmosphere) to the resulting mixture of Zn linoleate with the core and following stirring for 10 min at 240 °C. Isolation and purification of core/shell QDs were performed similarly to the procedure described in the synthesis of Cd_0.25_Zn_0.75_Se QDs. The weight of Cd_0.25_Zn_0.75_Se/ZnS 1 ML QDs after drying was 0.32 g.

#### 2.3.3. Synthesis of Core/Shell Cd_0.25_Zn_0.75_Se/ZnS 2 ML QDs

The procedure was similar to the previous synthesis. For 0.196 g of core Cd_0.25_Zn_0.75_Se, ZnO (0.00278 mol), LA (3.46 mL), ODE (7 mL), and a solution of (*Z*)-1-(octadec-9-enyl)-3-phenylthiourea (0.00139 mol) in OAm (0.6 mL) and ODE (0.8 mL) were taken. The weight of Cd_0.25_Zn_0.75_Se/ZnS 2 ML QDs after drying was 0.43 g.

#### 2.3.4. Synthesis of Core/Shell Cd_0.25_Zn_0.75_Se/ZnS 3 ML QDs

For 0.196 g of core Cd_0.25_Zn_0.75_Se, ZnO (0.00474 mol), LA (4.4 mL), ODE (9 mL), and a solution of (*Z*)-1-(octadec-9-enyl)-3-phenylthiourea (0.00237 mol) in OAm (1.0 mL) and ODE (1.0 mL) were taken. This solution was added in portions (0.3 mL/2 min). The weight of Cd_0.25_Zn_0.75_Se/ZnS 3 ML QDs after drying was 0.67 g.

#### 2.3.5. Synthesis of Core/Shell Cd_0.25_Zn_0.75_Se/ZnS 4 ML QDs

For 0.196 g of core Cd_0.25_Zn_0.75_Se, ZnO (0.00712 mol), LA (6.6 mL), ODE (14 mL), and a solution of (*Z*)-1-(octadec-9-enyl)-3-phenylthiourea (0.00356 mol) in OAm (1.0 mL) and ODE (0.5 mL) were taken. This solution was added in portions (0.3 mL/2 min). The weight of Cd_0.25_Zn_0.75_Se/ZnS 4 ML QDs after drying was 0.79 g.

#### 2.3.6. Synthesis of Core/Shell Cd_0.25_Zn_0.75_Se/ZnS 5 ML QDs

For 0.196 g of core Cd_0.25_Zn_0.75_Se, ZnO (0.00992 mol), LA (9.3 mL), ODE (20 mL), and a solution of (*Z*)-1-(octadec-9-enyl)-3-phenylthiourea (0.00496 mol) in OAm (2.0 mL) were taken. This solution was added in portions (0.4 mL/2 min). The weight of Cd_0.25_Zn_0.75_Se/ZnS 5 ML QDs after drying was 0.94 g.

### 2.4. Characterization

IR spectra in the region 50–450 cm^−1^ (resolution 2 cm^−1^) were recorded on a Vertex 70v FT-IR spectrometer (Bruker, Germany) using a single-bounce diamond ATR crystal. The room-temperature Raman spectra were measured by the Raman spectrophotometer MultiRam (Bruker, Germany). The YAG:Nd laser line (1064 nm) was used for excitation. X-ray diffraction patterns (XRD) were registered using the PANalytical EMPYREAN powder X-Ray diffractometer (ALMELO, Netherlands) with Cu-Kα radiation (λ = 1.5418 Å). Data were obtained across a 2θ range of 20–70° with a step size of 0.05°.

The Nuclear Magnetic Resonance (NMR) spectra were recorded from solutions in CDCl_3_ at 295 K on a Bruker Ascend^TM^ 500 spectrometer (equipped with Z-gradient 5 mm Prodigy cryoprobe) at frequencies of 500.13 MHz for ^1^H, 125.76 MHz for ^13^C{^1^H}, and 95.35 MHz for ^77^Se{^1^H}. The solutions were obtained by dissolving approximately 20–40 mg of each compound in 0.6 mL of the deuterated solvent. The values of ^1^H chemical shifts were calibrated to residual signals of CDCl_3_ (δ(^1^H) = 7.26 ppm). The values of ^13^C chemical shifts are referred to signals of CDCl_3_ (δ(^13^C) = 77.23 ppm). The values of ^77^Se chemical shifts are referred to Me_2_Se (δ(^77^Se) = 0.0 pm). Positive chemical shift values denote shifts to the higher frequencies relative to the standards.

The surface chemical composition was determined by X-ray photoelectron spectroscopy (XPS, ESCA 2SR, Scienta-Omicron) using a monochromatic Al Kα source (1486.6 eV). QDs were pressed into C tape for the XPS analysis, and the possible charging effects were compensated using an electron flood gun. The following sequence of spectra was recorded: survey, C *1s*, O *1s*, Cd *3d*, Zn *2p*, Se *3d*, S *2p*, and C *1s* again to verify the stability of the charge compensation as a function of time. The survey spectra were recorded at a pass energy of 150 eV, while the high-resolution spectra were recorded at a pass energy of 20 eV. The binding energy scale was referenced to adventitious carbon (284.8 eV) and the quantitative analysis was performed using sensitivity factors provided by the manufacturer.

Cd_0.25_Zn_0.75_Se (core) and Cd_0.25_Zn_0.75_Se/ZnS 1–5 ML (core/shell) QDs were predeposited onto lacey-carbon-coated (with/without graphene oxide layer) copper grids, and TEM, STEM, HRTEM, and EDS analyses and mapping were performed using a JEOL 2200FS transmission electron microscope (JEOL, Japan) equipped with a Centurio SDD-EDS detector. The TEM samples of QDs were prepared by dropping of QDs solution on a TEM copper grid with ~1 nm-thick graphene oxide on a lacey carbon membrane. To visualize the structure of the QDs with sufficient atomic resolution and contrast, high-resolution TEM imaging (HRTEM) was performed. The chemical composition of the single QD was carried out in a form of elemental maps using energy-dispersive X-ray (EDS) microanalysis in high-angle annular dark-field (HAADF) STEM mode using a Centurio EDS. In addition, energy-dispersive X-ray microanalysis of bulk nanoparticles samples (EDS) was carried out using the scanning electron microscope LYRA 3 (Tescan, Czech Republic) equipped with the EDS analyzer Aztec X-Max 20 (Oxford Instruments) at an acceleration voltage of 20 kV and with an FEI SUPER-X 4-quadrant windowless detector with a 120 mm^2^ total detection area and 0.7 sr solid angle. The sample was deposited onto a 3 nm carbon membrane copper grid.

The QDs optical properties were measured using a Fluorometer PTI QuantaMaster 400 (Horiba Scientific) to obtain PL data in the spectral range of 300–750 nm using an excitation wavelength of *λ* = 300–500 nm and UV-3600 (Shimadzu) spectrometer to obtain UV-VIS absorbance spectra in the spectral range of 200–700 nm. The PL lifetime measurements were performed using TCSPC accessories for a Fluorometer PTI QuantaMaster 400 with 362, 395, and 455 nm light pulse excitations and a pulse half-width of 0.8 ns produced by NanoLED-360, 375, and 455, respectively (Horiba Scientific). Photoluminescence decay kinetics curves were analyzed by PTI Felix GX software.

## 3. Results and Discussion

### 3.1. Synthesis of Core/Shell Cd_0.25_Zn_0.75_Se/ZnS (1–5 MLs) QDs

**Figure 1:**
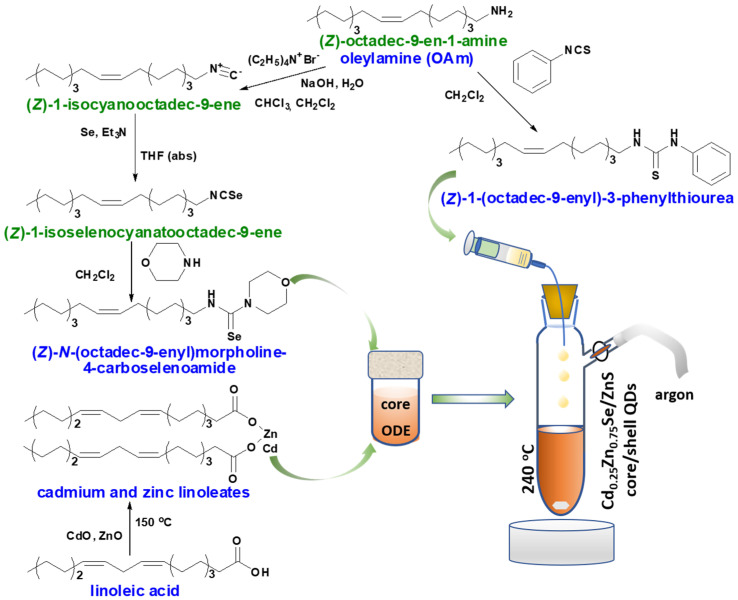
Schematic overview of the Cd_0.25_Zn_0.75_Se/ZnS (1–5 MLs) core/shell QDs synthesis.

The application of di- and trisubstituted thio- and selenoureas as effective, available, and environmentally safe sources of chalcogens has been presented previously [15,34,35,36]. The syntheses of (*Z*)-*N*-(octadec-9-enyl)morpholine-4-carboselenoamide and (*Z*)-1-(octadec-9-enyl)-3-phenylthiourea (Figure 1) proceeded without any complications and with good yield, which can only be limited by the purity of the reagents.

It was demonstrated that the formation of nanosized binary [35] and ternary sulfides [36] and selenides [15] begins with the formation of intermediate complexes of metal linoleates with substituted thio- or selenourea. The formed complex is unstable at high temperatures, which leads to its decomposition and initiates the onset of nucleation. Continuously forming nuclei grow by consuming the building material from the reaction medium. In parallel, the dissolution of smaller nuclei takes place, causing the homogeneity of the nanocrystals at the end of the reaction process. We presume that the creation of a shell on already formed and homogeneous nuclei occurs by a similar mechanism. To prevent the formation of nanocrystals grown from the shell material (i.e., ZnS), it is necessary to correlate the amounts of substances of all participants in the process. To determine the amount of the Cd_0.25_Zn_0.75_Se substance, the resulting nanomaterial was chemically decomposed, namely, the selenide ion was replaced by chloride. When Cd_0.25_Zn_0.75_Se QDs dried to constant weight were treated with 10% HCl, water-soluble metal chlorides and water-insoluble linoleic acid formed. During the further extraction, linoleic acid was fully transferred into diethyl ether. Thus, it was found that 0.196 g of nanomaterial contained 0.17 g of Cd_0.25_Zn_0.75_Se QDs.

**Figure 2:**
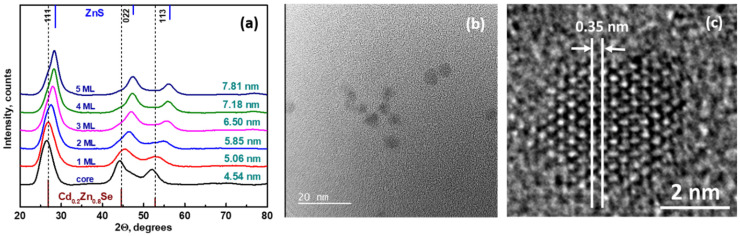
XRD patterns of Cd_0.25_Zn_0.75_Se/ZnS QDs with different shell thicknesses (**a**), TEM (**b**), and HRTEM (**c**) images of Cd_0.25_Zn_0.75_Se (core).

To develop a calculation model, we proceeded from empirical data, such as the average diameter (4.54 nm according to the TEM data, see Figure 2b) and the real composition of the synthesized spherical Cd_0.25_Zn_0.75_Se core, and also from theoretical assumptions, such as the thickness of ZnS 1 ML, which is 0.31 nm for a cubic structure [37]. Thus, taking the shell radius (*r_1_*) as a constant value, we can calculate the volume of the Cd_0.25_Zn_0.75_Se core (*V_1_*) and its mass (*m_1_*), taking the density as a sum of the parts of the densities of binary selenides *ρ_core_* = 5.4 × 10^6^ g/m^3^ (Equations (1) and (2)):

(1) $$V_{1}=\frac{4}{3}\;\pi \;{r_{1}}^{3\;}=48.97\;nm^{3}=48.97\cdot 10^{-27}\;m^{3}$$

(2) $$m_{1}=5.4\cdot 10^{6}\;\cdot 48.97\cdot 10^{-27}=2.64\cdot 10^{-19}\;g$$

Therefore, if *r_2_* = 0.31*x* + *r_1_*, where *x* is the number of monolayers, then at *x* = 1, *m* (ZnS 1 ML) will be 0.93 × 10^−19^ g (Equation (3)):

(3) $$m_{shell}=V_{shell}\cdot \;\rho_{ZnS}=\left(V_{\;}-V_{1}\right)\cdot 4.09\cdot 10^{6}=0.93\cdot \;10^{-19}\;g$$

where *V* is the volume of the Cd_0.25_Zn_0.75_Se/ZnS core/shell and *ρ_ZnS_* is the density of ZnS.

Taking the mass of the core as 100%, we can calculate the ratio of the masses of the core and the shell, which, in the case *x* = 1, will be 35.2%. In the synthesis of Cd_0.25_Zn_0.75_Se/ZnS (1–5 MLs) QDs, we took 0.196 g of the core (in terms of pure selenide 0.17 g):

(4) $$\upsilon ,\;\mathrm{mol}\;\left(\mathrm{ZnS}\right)=\frac{0.17\;\cdot 0.352}{M\left(\mathrm{ZnS}\right)}=\frac{0.06}{97.474}=6.15\cdot 10^{-4}\mathrm{mol}=\upsilon ,\;\mathrm{mol}\;\left(TU\right)$$

Using this approach, the amounts of (Z)-1-(octadec-9-enyl)-3-phenylthiourea (*TU*) taken to form 2, 3, 4, and 5 MLs were also calculated, respectively.

### 3.2. Morphology and Analytical Characterization of Cd_0.25_Zn_0.75_Se/ZnS (1–5 MLs) QDs

Figure 2a displays X-ray patterns for Cd_0.25_Zn_0.75_Se core/shell QDs with different thicknesses of the ZnS shell. The diffraction peaks for all samples lie between corresponding peaks for bulk cubic crystalline phases of Cd_0_._2_Zn_0_._8_Se (ICSD 98-019-2340) and ZnS (ICSD 98-005-3943) (the respective XRD patterns are given at the bottom and top of Figure 2a). The whole series of prepared QDs have broadened peaks, which indicate small particle sizes. The X-ray diagram of the Cd_0.25_Zn_0.75_Se core possesses a face-centered cubic structure with the space group *F*$\bar{4}$*3m*. The three prominent peaks at 2*θ* = 26.5, 44.3, and 52.2° are located between the respective peaks for bulk ZnSe (ICSD 98-016-7830) and CdSe (ICSD 98-018-7310), which suggests a successful formation of the alloyed crystal structure of Cd_0.25_Zn_0.75_Se QDs. Reflection with the highest intensity observed from Cd_0.25_Zn_0.75_Se QDs originated from the (111) crystallographic plane. No other peaks from Cd or Zn impurity phases were detected in the XRD patterns of Cd_0.25_Zn_0.75_Se QDs. The presented data are consistent with our previous report [15].

During the overcoating process with the ZnS shell, the crystal structure of the Cd_0.25_Zn_0.75_Se/ZnS QDs remains unchanged, i.e., zinc-blended crystalline structure. It is clearly visible that with an increase in the shell thickness, there is a gradual shift in the peak to larger 2*θ* angles, pointing to the formation of the ZnS-rich phase. Such shifts in the characteristic peaks further highlight the influence of the ZnS shell on the Cd_0.25_Zn_0.75_Se/ZnS core/shell QDs and agree well with the results from TEM measurements and optical properties.

The presented X-ray diffraction results are well-matched with the literature [38]. The lattice parameters of the core (5.7934 Å) and shell (4.9510 Å) and mean particle size were calculated from the Scherrer equation [34].

**Figure 3:**
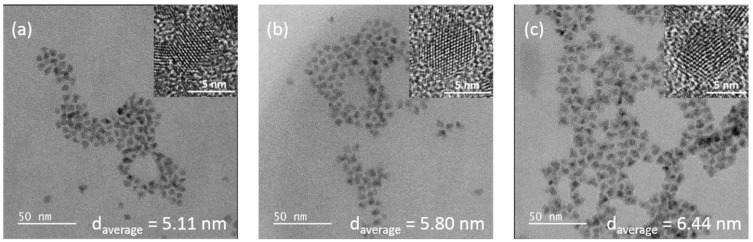
TEM and HRTEM (inset) images of Cd_0.25_Zn_0.75_Se/ZnS 1 ML (**a**), Cd_0.25_Zn_0.75_Se/ZnS 2 MLs (**b**), and Cd_0.25_Zn_0.75_Se/ZnS 3 MLs (**c**).

**Figure 4:**
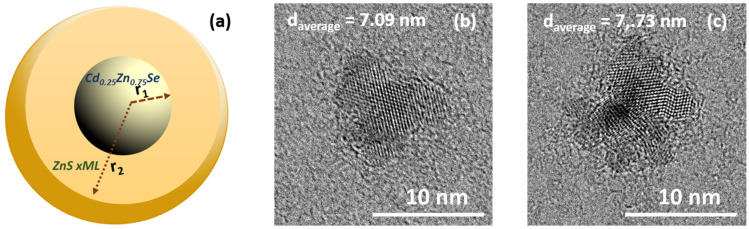
Theoretical model of core/shell quantum dot (**a**), HRTEM images of Cd_0.25_Zn_0.75_Se/ZnS 4 MLs (**b**), and Cd_0.25_Zn_0.75_Se/ZnS 5 MLs (**c**).

Figure 2b,c show TEM and HRTEM images of as-prepared Cd_0.25_Zn_0.75_Se QDs correspondingly. A dilute solution of QDs in chloroform was sonicated for 10 min and deposited on a TEM copper grid with an electron transparent membrane from graphene oxide. As can be seen from Figure 2b,c, the QDs were quite monodisperse and had a quasi-spherical shape. The interatomic distance in the core was 0.35 nm. Upon overcoating 1 and 2 MLs of ZnS shell on the surface of the Cd_0.25_Zn_0.75_Se QDs core, epitaxial growth of the ZnS shell was observed (Figure 3a,b). Onwards, with a ZnS 3 ML overcoating, partially epitaxial growth was noticed (Figure 3c). Further growth of the ZnS shell to 5 MLs exhibited nonepitaxial growth, which was confirmed by HRTEM (Figure 4b,c).

**Figure 5:**
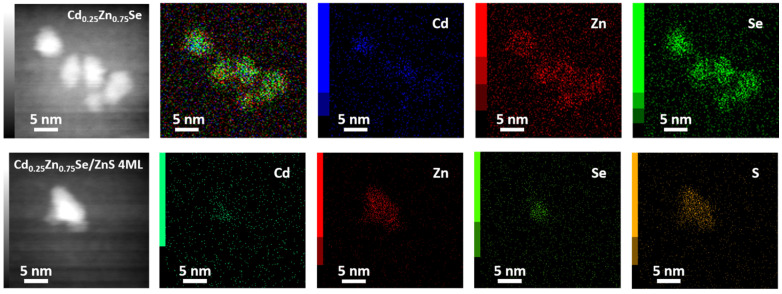
HAADF-STEM image and corresponding EDS elemental mapping of Cd, Zn, S, and Se of Cd_0.25_Zn_0.75_Se/ZnS core/shell QDs.

Figure 5 demonstrates HAADF-STEM imaging and corresponding energy dispersive X-ray spectroscopy (EDS) mapping for the core (Cd_0.25_Zn_0.75_Se) and for core/shell QDs (Cd_0.25_Zn_0.75_Se/ZnS 4 MLs). It can be seen from this figure that in the core, all elements (Cd, Zn, and Se) are present in the entire volume of the QD. At the same time, for Cd_0.25_Zn_0.75_Se/ZnS 4 ML QDs, the presence of Cd and Se is observed only in the core, while Zn and S are distributed in the entire volume of the QD. These results revealed that Cd, Zn, and Se are distributed homogeneously in QDs. There is no visual element aggregation or separation. The intensity of these signals is in good agreement with the nominal compositions of QDs.

The elemental analysis data of the QDs measured by the EDS and XPS analysis are summarized in Table 1. According to the data, the ratio of elements in the core material is consistent with the nominal composition. When the shell grows, the ratios of metals and chalcogens change according to the calculated amounts of substances taken into the synthesis. The ratios of the elements determined by XPS analysis differ from the nominal ones while corresponding to the QDs sizes. Signals corresponding to carbon and oxygen were also detected. These signals are attributed to metal linoleates present on the surface of the QDs as a protective shell. Traces of nitrogen, also found in the EDS spectrum, belong to the *co*-ligand oleylamine.

**Figure 6:**
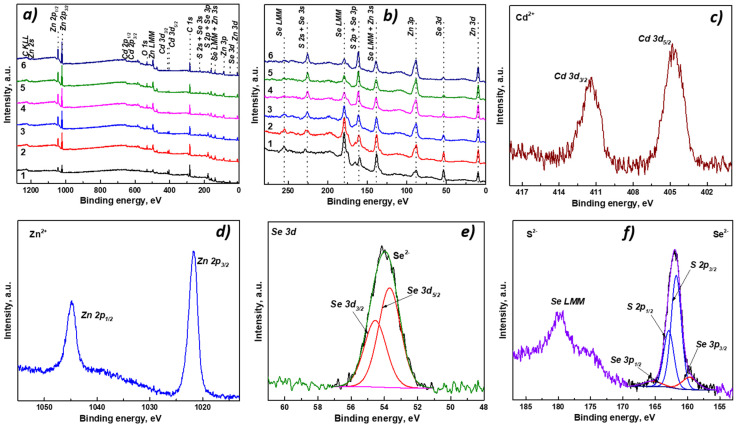
XPS survey spectra of core/shell Cd_0.25_Zn_0.75_Se/ZnS QDs with different shell thicknesses (1—Cd_0.25_Zn_0.75_Se core, 2—ZnS 1 ML, 3—ZnS 2 MLs, 4—ZnS 3 MLs, 5—ZnS 4 MLs, 6—ZnS 5 MLs) (**a**,**b**); chemical state analysis of Cd *3d* (**c**), Zn *2p* (**d**), and Se *3d* (**e**), and strong overlap between S *2p* and Se *3p* (**f**).

The surface chemical state of Cd_0.25_Zn_0.75_Se/ZnS QDs was studied by XPS. The presence of C, O, Cd, Zn, Se, and S was confirmed through the survey spectra displayed in Figure 6a. Carbon and oxygen signals are due to the organic ligands from the precursors. Cd, Zn, and Se peaks correspond to the core of QDs; however, Zn is also part of the shell, as well as sulfur. In addition, the change in intensity of the Zn signal of the core sample with respect to the core/shell samples was observed. This was due to the fact that by adding the ZnS, a greater amount of zinc would be detected. This effect is more visible with sulfur and selenium, as the sulfur signal (S *2p*) increases as more ZnS is added in the QDs, and the selenium signal (Se *3d*) consequently decreases, as presented in Figure 6b. Additionally, Table 1 displays the results of the elemental composition of the samples and it demonstrates the aforementioned trend.

Figure 6c displays the Cd 3*d* high-resolution (HR) spectrum of core/shell QDs, where its spin–orbit splitting Cd 3*d_5/2_*/Cd 3*d_3/2_* signals are centered at ~404.8 eV/411.5 eV, assigned to the Cd^2+^ state related to Cd-Se bonds [39,40]. The Zn 2*p* HR spectrum is illustrated in Figure 6d, and its doublet, Zn 2*p_3/2_*/Zn 2*p_1/2_*, appears at ~1021.8 eV/ 1044.8 eV and suggests the presence of Zn^2+^ [41,42]. In the Se 3*d* HR spectrum displayed in Figure 6e, the presence of Se^2−^ species was found, as the spin–orbit signal Se 3*d_5/2_*/Se 3*d_3/2_* is located at ~53.8 eV/54.7 eV [39,40]. In Figure 6f, a strong overlap between the S 2*p* and Se 3*p* signals is evidenced. The spectrum was deconvoluted with four components, and the most intense pair corresponds to the spin–orbit splitting of sulfur S 2*p_3/2_*/S 2*p_1/2_*, centered at ~161.7 eV/162. 9 eV, which is attributed to S^2−^ species, related to the Zn-S bond from the shell [42,43]. The less intense doublet Se 3*p_3/2_*/Se 3*p_1/2_* located at ~160.2 eV/166.0 eV confirms the presence of Se^2-^ found in Se 3*d* signal [39].

**Figure 7:**
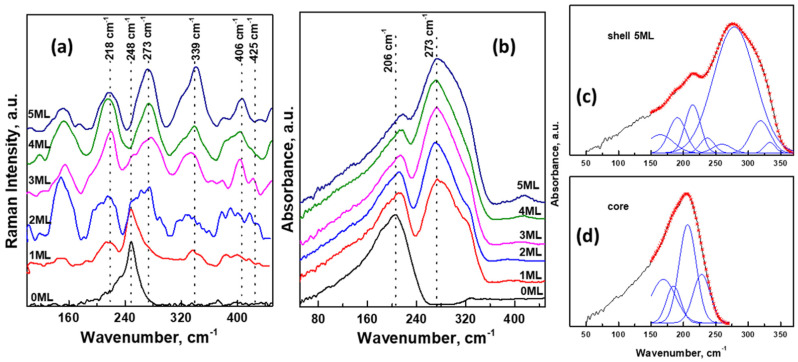
Raman (**a**) and ATR (**b**) spectra of Cd_0.25_Zn_0.75_Se/ZnS (0–5 MLs) in the region 50–450 cm^−1^; the decomposition of ATR spectra of Cd_0.25_Zn_0.75_Se/ZnS (5 MLs) (**c**) and core (**d**).

The Raman spectra of Cd_0.25_Zn_0.75_Se/ZnS core/shell QDs series with different shell thicknesses are presented in Figure 7a. In the case of the core Cd_0.25_Zn_0.75_Se sample, the only dominant band with maxima 248 cm^−1^ can be observed in the Raman spectra. This band can be attributed to longitudinal optical (LO) phonons in cubic Cd_0.25_Zn_0.75_Se QDs [44,45]. When the core is encased within a ZnS shell, the new peaks attributed to the shell appear. The intensity of the band at 248 cm^−1^ gradually decreases with the growing thickness of the ZnS shell, and for the Cd_0.25_Zn_0.75_Se/ZnS (5 MLs) QDs, only a very weak shoulder is visible. The new bands at 218 cm^−1^, 273 cm^−1^, and 339 cm^−1^ are associated with longitudinal acoustical (LA) overtones and transverse optical (TO) and longitudinal optical (LO) phonon modes, and the less intense bands at 406 cm^−1^ and 425 cm^−1^ can be assigned to the sum (TO + LA) and (LO + TA) combination bands [35,46]. These results are good confirmation of the gradual growth of ZnS layers around the Cd_0.25_Zn_0.75_Se core.

ATR spectra of Cd_0.25_Zn_0.75_Se/ZnS core/shell QDs in the region 50–450 cm^−1^ are demonstrated in Figure 7b. In this region, the main absorbance bands of inorganic nanoparticles are located. The ATR spectrum of pure Cd_0.25_Zn_0.75_Se QDs contains a relatively strong band with maxima at 206 cm^−1^ in the spectral region of 50–250 cm^−1^. The growth of the ZnS shell causes the appearance of a new intensive band with maxima at 273 cm^−1^ in the region between 230 cm^−1^ and 360 cm^−1^. The intensity of this band enhances with increasing layer thickness, while the intensity of the first band (at 206 cm^−1^) remains the same. ATR spectra were deconvoluted into several separate individual bands (as an example, decomposed spectra of core Cd_0.25_Zn_0.75_Se and Cd_0.25_Zn_0.75_Se/ZnS (5 MLs) QDs are illustrated in Figure 7c,d). The intensity of absorbance bands in the region of lower wavenumbers was influenced by the background due to the lower sensitivity of the detector and ATR crystal in this area; therefore, the figures display deconvolutions only in the area of the main vibration bands (150–370 cm^−1^). In the case of pure Cd_0.25_Zn_0.75_Se QDs (Figure 7d), the spectra were decomposed into four bands: strong band at 206 cm^−1^ and medium bands at 168 cm^−1^, 184 cm^−1^, and 225 cm^−1^. These frequencies are in good agreement with the frequencies of vibration modes obtained from the reflection spectra of Cd_x_Zn_1−x_Se alloys [47]. The strongest band can be assigned to TO vibrations in Cd_0.25_Zn_0.75_Se QDs. In the case of core/shell samples (Figure 7c), the spectra were decomposed into 8 bands. Likely, the first four bands are again related to vibrations in the Cd_0.25_Zn_0.75_Se core. The second four bands at 259 cm^−1^, 278 cm^−1^, 318 cm^−1^, and 333 cm^−1^ can be assigned to vibrations in the ZnS shell with cubic structure [48,49]. These results are in good agreement with those obtained from the Raman spectra of our samples.

### 3.3. Optical Properties of Cd_0.25_Zn_0.75_Se/ZnS (1–5 MLs) QDs

**Figure 8:**
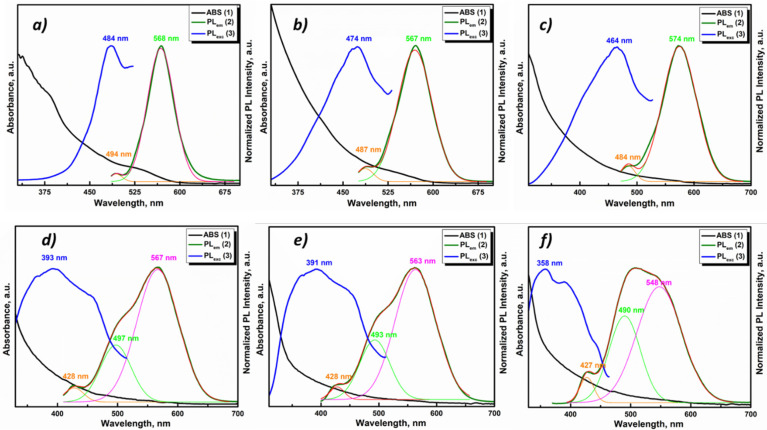
Normalized ABS, PL excitation, and PL emission spectra of core/shell Cd_0.25_Zn_0.75_Se/ZnS QDs with different shell thicknesses: (**a**) Cd_0.25_Zn_0.75_Se core, (**b**) ZnS 1 ML, (**c**) ZnS 2 MLs, (**d**) ZnS 3 MLs, (**e**) ZnS 4 MLs, and (**f**) ZnS 5 MLs.

One of the main goals of coating QDs with a thin layer of nanomaterial with different compositions is to change their optical characteristics. It is known that with the appropriate selection of the shell material, it is possible to achieve a manifold rise in the PL quantum yield and/or desired wavelength shift [50]. In the study of optical properties of the synthesized core/shell Cd_0.25_Zn_0.75_Se/ZnS (0–5 MLs) QDs, it was found that a change in the excitation wavelength affects only the intensity of the emission signal, without any variations in their form and position. The given tendency persists for each of the studied samples. Therefore, for the assessment and comparative analysis of emission signals, solutions of Cd_0.25_Zn_0.75_Se/ZnS (0–5 MLs) QDs were excited by light at λ_max_ so the intensity of the emission signal was maximum. Thus, the ratio of the intensities of the main and additional photoluminescence emission peaks was retained for all core/shell Cd_0.25_Zn_0.75_Se/ZnS QDs. Figure 8 displays the absorbance (black curves), excitation (blue), and emission (dark green) spectra of Cd_0.25_Zn_0.75_Se/ZnS (1–5 MLs) QDs. As follows from Figure 8, an increase in the number of ZnS monolayers in the Cd_0.25_Zn_0.75_Se QDs shell affected the spectra of all abovementioned characteristics.

In the absorbance (ABS) spectrum of Cd_0.25_Zn_0.75_Se QDs, there is a weak peak in the region of 530–540 nm (Figure 8a), which is caused by the absorbance of an exciton. At the moment of the ZnS shell increment in the Cd_0.25_Zn_0.75_Se core, this peak smooths out (1 ML ZnS Figure 8b), and after, completely disappears from the ABS spectra of Cd_0.25_Zn_0.75_Se/ZnS QDs (2–5 MLs) QDs (Figure 8c–f). It can be caused by a growing size of Cd_0.25_Zn_0.75_Se/ZnS QDs (1–5 MLs) QDs, as the exciton intensity decreases with the QD’s size. Coating Cd_0.25_Zn_0.75_Se QDs with ZnS 1 ML enlarges the size of QDs by ≈0.6 nm in diameter, which is 13% for Cd_0.25_Zn_0.75_Se QDs 4.54 nm in size. In addition, the band gap values of Cd_0.25_Zn_0.75_Se/ZnS QDs (1–5 MLs) were estimated from the absorbance spectra using the Tauc equation [51]. The calculation results are presented in Table 2.

**Figure 9:**
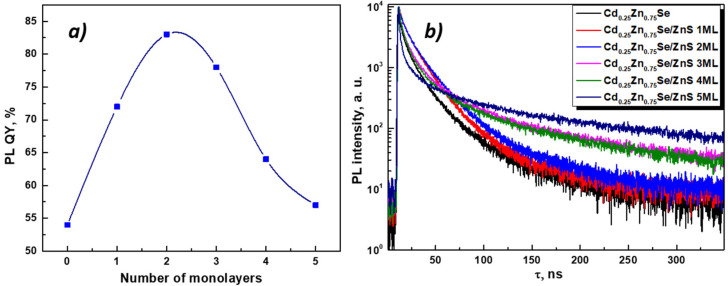
PL QY dependence on the shell thickness (**a**) and PL decay kinetic curves (**b**) of core/shell Cd_0.25_Zn_0.75_Se/ZnS QDs.

Following the increase in the number of monolayers on the surface of the core, significant changes in the PL excitation and emission spectra can be seen. For a more informative presentation, the emission spectra were decomposed into Gaussians. The excitation and emission spectra of the Cd_0.25_Zn_0.75_Se core are represented by narrow Gaussian bands (Figure 8a). A small, low-intensity peak in the blue region of the spectrum can be caused by scattering and reabsorption of the sample photoluminescence due to the high excitation density. When ZnS 1 (Figure 8b) and 2 MLs (Figure 8c) were incremented to the Cd_0.25_Zn_0.75_Se core, the PL excitation spectra noticeably broadened, and their maximum shifted to the blue region (see Table 2). In addition, there is some broadening in the emission spectra with a shift in the maxima toward the red region of the spectrum (*λ_em_* = 567 nm and *λ_em_* = 574 nm for Cd_0.25_Zn_0.75_Se/ZnS QDs 1 ML and 2 MLs, respectively). When the ZnS 1 ML grows on the Cd_0.25_Zn_0.75_Se core, PL QY rises from 54% (for the Cd_0.25_Zn_0.75_Se core) to 83% (for the Cd_0.25_Zn_0.75_Se/ZnS 2 MLs) (Figure 9a). With a subsequent augmentation of the shell thickness to 3–5 MLs, additional components appear in the PL excitation and emission spectra, and their maxima shift toward the high-energy spectral region. In addition, with an increase in the number of ZnS MLs, the intensity of the main PL component (*λ_em_* = 567 nm, 563 nm, and 548 nm for Cd_0.25_Zn_0.75_Se/ZnS 3 ML, 4 ML, and 5 ML QDs, respectively) decreases, while the additional PL component that appears (*λ_em_* = 497 nm, 493 nm, and 490 nm for Cd_0.25_Zn_0.75_Se/ZnS 3 ML, 4 ML, and 5 ML QDs, respectively) becomes dominant (Figure 8d–f). An increase in the thickness of the Cd_0.25_Zn_0.75_Se/ZnS QDs shell from 3 MLs and more has a strong effect on the PL intensity. For Cd_0.25_Zn_0.75_Se/ZnS 3 ML QDs, the PL QY decreases to 78%, and with subsequent growth in the number of ZnS MLs up to 5, the PL QY drops to 57% (Figure 9a). Such changes in the PL spectra of Cd_0.25_Zn_0.75_Se/ZnS QDs with rising shell thickness were caused by a change in the parameters of its growth on the surface of the Cd_0.25_Zn_0.75_Se core. On TEM images (Figure 3a,b), it was clearly visible that with increment in the ZnS 1 and 2 MLs to the Cd_0.25_Zn_0.75_Se core, the shell grows epitaxially. This means that the first two ZnS MLs grow evenly distributed over the surface of the core, “healing” most of the surface defects. This “healing” of defects leads to an increase in the PL intensity, almost without affecting the shape of the spectrum. The growth in the 4 or 5 MLs no longer occurs epitaxially to the core (Figure 4b,c). As the layer thickness is comparable with the crystal lattice parameter, and the further growing ZnS layer no longer interacts with the core, a ZnS monolayer increments on the surface with a lot of defects [50,52]. Increasing the shell thickness also leads to an enhancement in the volume/mass ratio of the shell and core. As can be seen from Figure 8d, an additional component (*λ_em_* = 497 nm) appears in the PL spectrum of Cd_0.25_Zn_0.75_Se/ZnS 3 ML QDs, which is induced by the luminescence of the shell. Consequently, the shell no longer amplifies the PL of the core, but already has its own PL, which means that it will absorb the exciting light.

As mentioned above, the number of surface defects decreases with epitaxial growth and increases with nonepitaxial growth. To confirm this statement, the quenching PL kinetics curves of core/shell Cd_0.25_Zn_0.75_Se/ZnS QDs were measured (Figure 9b). The quenching kinetics of a single crystal without defects consists of one exponential component. However, the surface of nanocrystals is characterized by many nonpassivated states (defects), which provide additional (radiative and nonradiative) relaxation paths of charge carriers. Due to that, the PL quenching kinetics of nanosized crystals is multi-exponential [53]. A change in the number of surface defects leads to a change in the PL quenching kinetics curves. The quenching kinetics curves measured for PL core/shell Cd_0.25_Zn_0.75_Se/ZnS QDs are multi-exponential. They were fitted using Equation (5):

(5) $$I\left(t\right)=\overset{t}{\underset{0}{\int }}IRF\left(t^{\prime }\right)\left(C+\overset{3}{\underset{i=1}{\sum }}A_{i}\exp\left(-\frac{t-t^{\prime }}{\tau_{i}}\right)\right)dt^{\prime }$$

where *IRF* is the instrument response function, and *τ_i_* and *A_i_* are the PL decay time and amplitudes components, respectively. The average lifetime is calculated using Equation (6):

(6) $$\tau_{avg}=\frac{\sum_{i=1}^{3}A_{i}\tau_{i}}{\sum_{i=1}^{3}A_{i}}$$

As follows from Figure 9b, when the ZnS 1 and 2 MLs were deposited onto the Cd_0.25_Zn_0.75_Se core, the quenching kinetics curves were slightly aligned (red and blue curves, respectively). This was also confirmed by the fitting results of the PL quenching kinetic curves of core/shell Cd_0.25_Zn_0.75_Se/ZnS QDs presented in Table 2. When the Cd_0.25_Zn_0.75_Se core is covered with one or two ZnS MLs, the component with *τ* ≈ 11 ns becomes dominant. At the same time, for the Cd_0.25_Zn_0.75_Se core, the main contribution is made by the fastest component (*τ* = 1.3 ns). According to other authors, the exciton lifetime in CdZnSe is in the range of 7–15 ns [3,54]. The presence of a fast PL quenching component is caused by the interaction of an exciton with energetically deep surface defects and its nonradiative relaxation [53]. Reducing the contribution of this component indicates a decrease in the number of defects and, as a result, an enhancement in the PL intensity. With the growth in the shell thickness to ZnS 3 MLs, the main contribution is again made by the fast component. It should also be noted that further growth in the number of ZnS MLs leads to a reduction in this component and a significant increase in its contribution (*τ* = 2.1 ns and *A* = 64%, and *τ* = 1.7 ns and *A* = 71% for ZnS 3 and 5 MLs, respectively). Last but not least, with a shell growth from ZnS 3 MLs, the last and slowest time component of the quenching kinetics of core/shell Cd_0.25_Zn_0.75_Se/ZnS QDs rises from *τ* = 30.3 ns for ZnS 2 MLs to *τ* = 69.9 ns for ZnS 3 MLs and *τ* = 86.1 ns for ZnS 5 MLs. The gain of the slow component indicates an augmentation in the interaction of charge carriers with small surface defects [53]. From the above, it follows that the growth of the ZnS shell thickness for Cd_0.25_Zn_0.75_Se/ZnS QDs by more than two monolayers leads to negative consequences, namely, an increase in the number of surface defects and a decrease in the PL core/shell Cd_0.25_Zn_0.75_Se/ZnS QDs intensity.

## 4. Conclusions

To conclude, we have presented the employment of an efficient and highly controlled methodology for growing the binary ZnS shell on ternary Cd_0.25_Zn_0.75_Se quasi-spherical QDs, which can be prepared on a multigram scale (e.g., 3.5 g per one batch). The method is based on the calculated volume and mass ratios of the core to the shell. This calculation allowed us to minimize the required materials to the shell growth of a desired thickness without the formation of independent ZnS crystals. It has also been demonstrated that substituted selenourea (i.e., (*Z*)-*N*-(octadec-9-enyl)morpholine-4-carboselenoamide), taken as a new source of selenium, was successfully combined with the already presented substituted thiourea (i.e., (*Z*)-1-(octadec-9-enyl)-3-phenylthiourea) within the synthesis of core/shell Cd_0.25_Zn_0.75_Se/ZnS QDs. Raman and FT-IR spectral studies, as well as EDS and XPS analysis of the core/shell Cd_0.25_Zn_0.75_Se/ZnS QDs, confirmed full accordance with the declared compositions. According to the TEM and optical studies, the 1 and 2 ML shells grew epitaxially to the core, the 3 ML shell grew partially epitaxially to the core, and a further increment in ZnS was characterized by nonepitaxial shell growth. Investigation of the PL properties of core/shell Cd_0.25_Zn_0.75_Se/ZnS 1–5 ML QDs revealed the PL QY considerable magnification (up to 83%) with the increment of 1 and 2 MLs of ZnS, which indicates the inhibition of nanocrystal’s surface defects. With a further shell thickness rise, new defects appeared, which led to a decrease in the PL QY. According to the experimental data, the 2 ML ZnS shell is optimal from the point of view of the morphology and optical properties of this nanomaterial. The method presented here certainly contributes to the development of further research and, due to its simplicity and accuracy, can be transferred to an industrial scale.

## Figures and Tables

**Table 1:** Elemental composition of Cd_0.25_Zn_0.75_Se/ZnS core/shell QDs measured by EDS * and XPS ** technique.

Composition	EDS					XPS			
	Cd, at.%	Zn, at.%	Se, at.%	S, at.%		Cd, at.%	Zn, at.%	Se, at.%	S, at.%
Cd_0.25_Zn_0.75_Se	14.7	41.2	44.1	-		16.3	48.2	35.6	-
Cd_0.25_Zn_0.75_Se/ZnS 1 ML	7.4	48.1	23.6	20.9		7.5	53.4	15.5	23.7
Cd_0.25_Zn_0.75_Se/ZnS 2 MLs	5.8	48.5	15.9	29.8		4.3	54.5	10.4	30.9
Cd_0.25_Zn_0.75_Se/ZnS 3 MLs	3.5	51.2	11.1	34.2		2.4	55.2	6.1	36.8
Cd_0.25_Zn_0.75_Se/ZnS 4 MLs	2.5	52.3	8.1	37.1		1.6	55.9	4.0	38.5
Cd_0.25_Zn_0.75_Se/ZnS 5 MLs	2.1	52.7	6.6	38.6		1.2	55.6	3.6	39.7

* Theoretical error value for EDS is 1% ** Theoretical error value for XPS is 0.1%.

**Table 2:** Optical properties of core/shell Cd_0.25_Zn_0.75_Se/ZnS QDs and parameters of PL decay curves evaluation. Peak maximum in the PLE spectra (*λ_exc_*), peak maximum in the PL spectra (*λ_em_*), Stokes shift and band gap (*E_g_*); fitting results: time constants *τ_1_, τ_2_*, *τ_3_*; amplitude components *A_1_*, *A_2_* and *A_3_*; average lifetime *τ_avg_*.

Number of Monolayers	Optical Parameters					Fitting Results of the PL Decay Curves						
	λ_exc_, nm	λ_em_, nm	QY,%	E_g_, eV		τ_1_, ns	τ_2_, ns	τ_3_, ns	A_1_	A_1_	A_1_	τ_avg_, ns
0	484	569	54	2.96		1.3	11.4	44.4	0.72	0.25	0.03	4.95
1	474	571	72	3.59		1.4	11.0	26.8	0.42	0.45	0.13	9.11
2	464	574	83	3.58		1.6	11.6	30.3	0.46	0.40	0.14	9.56
3	393	568	78	3.08		2.1	14.1	69.9	0.64	0.34	0.02	7.31
4	391	563	64	3.69		1.9	13.8	63.3	0.67	0.32	0.01	6.61
5	358	507	57	3.55		1.7	13.1	86.1	0.71	0.28	0.01	5.80
